# Clinical evaluation of non-invasive prenatal screening in 32,394 pregnancies from Changzhi maternal and child health care hospital of Shanxi China

**DOI:** 10.5937/jomb0-33513

**Published:** 2022-07-29

**Authors:** XiaoZe Li, LiHong Wang, ZeRong Yao, FangYing Ruan, ZhiPeng Hu, WenXia Song

**Affiliations:** 1 Changzhi Maternal and Child Health Care Hospital Affiliated Hospital of Changzhi Medical College, Department of Medical Genetic, Changzhi City, Shanxi Province, China

**Keywords:** NIPS, trisomy, false positive, true positive, Z score, SNPs variants, NIPS, trizomija, lažno pozitivan, istinski pozitivan, Z skor, SNP varijante

## Abstract

**Background:**

Non-invasive prenatal screening (NIPS) is a highly sensitive and specific screening test to detect fetal chromosomal abnormalities. The primary objective of this study was to evaluate the NIPS as an effective method for prenatal detection of aneuploidies in both high-risk and low-risk pregnancies.

**Methods:**

In current study, we performed NIPS in 32,394 pregnancies, out of which results were available in 32,361 (99.9%) of them. Illumina sequencing was performed for NIPS screening. Hypothesis Z test was used to classify fetal autosomal aneuploidy of T21, T18, and T13. Karyotyping was performed to determine the true negative and true positive NIPS results.

**Results:**

Among the 32,361 confirmed samples, 164 cases had positive results and 32197 cases had negative results. Of these positive cases, 116 cases were trisomy 21, 34 cases were trisomy 18 and 14 cases were trisomy 13. No false negative results were found in this cohort. The overall sensitivity and specificity were 100% and 99.91%, respectively. There was no significant difference in test performance between the 7,316 high-risk and 25,045 low-risk pregnancies, (sensitivity, 100% vs 100% (P>0.05); specificity, 99.96% vs 99.95% (P > 0.05)). Factors contributing to false-positive results included fetal copy number variants (CNVs), fetal mosaicism and typically producing Z scores between 3 and 4. Moreover, we analyzed NIPS wholegenome sequencing to investigate the Single Nucleotide Polymorphisms (SNPs) associations with drug response or risk of disease. As compare to the 1000g East Asian genome data, the results revealed a significant difference in 7,285,418 SNPs variants of Shanxi pregnant women including 19,293 clinvar recorded variants and 7,266,125 non-clinvar recorded.

**Conclusions:**

Our findings showed that NIPS was an effective assay that may be applied as routine screening for fetal trisomies in the prenatal setting. In addition, this study also provides an accurate assessment of significant differences in 7,285,418 SNPs variants in Shanxi pregnant women that were previously unavailable to clinicians in Shanxi population.

## Abstract (sr)

**Uvod:**

Neinvazivni prenatalni skrining (NIPS) je veoma osetljiv i specifičan skrining test za otkrivanje fetalnih hromozomskih abnormalnosti. Primarni cilj ove studije bio je da se proceni NIPS kao efikasan metod za prenatalno otkrivanje aneuploidije u trudnoćama visokog i niskog rizika.

**Metode:**

U trenutnoj studiji, NIPS smo uradili u 32.394 trudnoće, od kojih su rezultati bili dostupni u 32.361 (99,9%) trudnoća. Illumina sekvenciranje je izvršeno za NIPS skrining. Z test hipoteze je korišćen za klasifikaciju fetalne autozomne aneuploidije T21, T18 i T13. Kariotipizacija je urađena da bi se utvrdili pravi negativni i istinski pozitivni rezultati NIPS.

**Rezultati:**

Među 32.361 potvrđenim uzorkom, 164 slučaja je imalo pozitivne rezultate, a 32.197 slučajeva je imalo negativne rezultate. Od ovih pozitivnih slučajeva, 116 slučajeva je bilo trizomija 21,34 slučaja trizomija 18 i 14 slučajeva trizomija 13. Nisu pronađeni lažno negativni rezultati u ovoj kohorti. Ukupna osetljivost i specifičnost bile su 100% i 99,91%, respektivno. Nije bilo značajne razlike u performansama testa između 7.316 visokorizičnih i 25.045 trudnoća sa niskim rizikom, (osetljivost, 100% naspram 100% (P>0,05); specifičnost, 99,96% naspram 99,95% (P > 0,05)). Faktori koji su doprineli lažno pozitivnim rezultatima uključivali su varijante broja kopija fetusa (CNV), fetalni mozaicizam i tipično stvaranje Z rezultata između 3 i 4. Štaviše, analizirali smo sekvenciranje celog genoma NIPS da bismo istražili povezanost polimorfizama jednog nukleotida (SNP) sa odgovorom na lek. ili rizik od bolesti. U poređenju sa podacima o genomu istočne Azije od 1000 g, rezultati su otkrili značajnu razliku u 7.285.418 varijanti SNP-a kod trudnica u Shanki-u, uključujući 19.293 zabeležene varijante klinvara i 7.266.125 zabeleženih ne-clinvara.

**Zaključak:**

Naši nalazi su pokazali da je NIPS bio efikasan test koji se može primeniti kao rutinski skrining za fetalne trisomije u prenatalnom okruženju. Pored toga, ova studija takođe pruža tačnu procenu značajnih razlika u 7.285.418 varijanti SNP-a kod trudnica u Shanki-u koje su ranije bile nedostupne kliničarima u populaciji Shanki-a.

## Introduction

China has the largest number of birth defects in the world, with about 900,000 new cases of birth defects every year [1], including about 240,000 cases of chromosomal abnormalities, most of which cannot be cured till today [2]. In 1997, Lo et al. [3] discovered the presence of fetal cell free DNA in maternal peripheral (also named cfDNA). The cfDNA in maternal plasma grows in concentration with the gestational age, and it also harbors genomic information of the fetus. When a fetus has an abnormal number of chromosomes (aneuploidy), the cfDNA ratio regarding that chromosome will be altered [4]. However, the concentration of cfDNA in the maternal plasma circulation may vary widely [5] [6], ranging from 4% to over 30% [7]. Thus, advanced technologies such as digital polymerase chain reaction or massively parallel sequencing have been used to study cfDNA in maternal blood, differentiate fetal DNA from maternal DNA, and detect fetal chromosomal abnormalities [8]. These discoveries made non-invasive prenatal screening (NIPS) of fetal aneuploidies a clinical reality and led to a new era of non-invasive prenatal screening with high sensitivity and specificity in multiple clinical centers [9] [10]. NIPS had been commercialized in China since May 2010, and the clinicians showed strong interest and attempted to adopt the technology for detection of fetal aneuploidies [11]. Find Gene NIPS is one of the earliest NIPS assays developed in China. In November 2019, based on retrospective clinical trials conducted in multiple clinical centers, FindGene NIPS got approval from the National Medical Products Administration for screening fetal trisomies (T) 21, 18, and 13 in China. Moreover, sufficiently large NIPS samples also indicate population genetics databases such as SNPs and allele frequency, genomic locations, and functional annotations. Single Nucleotide Polymorphisms (SNPs) are known to contribute to variation in various diseases and drugs responses. The primary objective of this study was to evaluate the NIPS as an effective method for prenatal detection of aneuploidies in both high-risk and low-risk pregnancies. Moreover, we also investigated the SNPs induced risk of disease based on 32,394 samples obtained from the Changzhi Maternal and Child Health Care Hospital, and SNPs induced variation in drug response.

## Materials and methods

### Study design

This study was approved by the institutional review board of the Changzhi Maternal and Child Health Care Hospital. This was a prospective, largescale, blinded cohort study conducted from 11 October 2016 to 26 June 2019. Only the patients with written consent were included in this study. The inclusion criteria for participants were: pregnant women, at least 18 years old, and a gestational age of 11 to 30 weeks. The women who were excluded included those: (i) who had recent stem cell therapy or immune therapy; (ii) who had cancer diagnosed; (iii) who had known chromosome abnormalities or whose partners had known aneuploidy.

### NIPS screening

Five milliliter peripheral blood of pregnant women was collected by EDTA anticoagulant tube and delivered to the laboratory within 6 hr. of collection. The plasma was isolated from the peripheral blood by two-step centrifugation including at 1,600 × g for 10 min and then at 16,000 ×g for 10 min at 4°C. After that the samples were frozen and delivered to Shanghai Findgene clinical laboratories (Shanghai, China) at which plasma was prepared for library construction, quality control and pooling. The plasma DNA was extracted from 600 μL plasma of each sample using Circulating Nucleic Acid Kit from Findgene (Chengdu Findgene Medical Equipment Co., Ltd). The detailed general technical procedure was described previously [12]. The prepared library was quantified using real time PCR, 96 barcoded libraries which were titrated and evenly pooled and sequenced with 36-cycles single-end multiplex sequencing strategy on an Illumina Next Seq. 500 platform (Illumina, USA) [13].

Hypothesis Z test was used to classify fetal autosomal aneuploidy of T21, T18, and T13. An approach integrating dynamic GC reference library [14] and LOESS (locally weighted scatterplot smoothing) regression [15] was applied to correct the GC bias before Z test. Integrating maternal copy number variant (CNV) and fetal fraction correction [2] were applied to correct the Z score after Z test. The sequences of each sample were mapped to the reference genome of human and Z scores were calculated for each chromosome [16] [17]. Z ≥ 3 would represent a chromosomal aneuploidy and -3.0 < Z < 3.0 would represent a chromosomal euploid. Fetal fraction of male fetuses was calculated based on the Y chromosome fraction. Re-sequencing, retesting and resampling was applied when samples did not meet quality control (QC) standards. Re-sequencing was performed on samples with insufficient unique reads only. Common reasons for retested sample included insufficient cfDNA content after library preparation quantification and unsuitable GC content to the reference or Z score chaos (Z ≥ 3 or Z ≤ -3) for more than five chromosomes. Samples with hemolysis, gestational age of less than 12 weeks, insufficient fetal fraction, and repeating Z score chaos would be resampled. Only one chance of resampling was allowed.

### Statistical analysis

Statistical analysis between the different groups was performed using a chi square test or Fisher’sexact test, and *P* values of <0.05 were considered statistically significant.

### Clinical follow-up

Patients with negative NIPS screening resultswere advised for regular prenatal care; genetic counseling was provided if routine ultrasound examinationshowed abnormalities. Karyotyping was performedfor all NIPS screening samples. On the basis of karyotyping a negative NIPS result was considered as atrue negative if the prenatal or neonatal karyotypingresults were normal, or if the neonate looked phenotypically normal. By contrast, a negative NIPS resultwas defined as a false negative if the prenatal orneonatal showed aneuploidy in karyotyping, or if thenewborn was phenotypically abnormal. Pregnantwomen with positive NIPS results were recommendedfor confirmatory invasive prenatal diagnosis likeamniocentesis. The positive NIPS result was definedas true positive upon karyotyping invasive confirmation or clinical follow-up results. The false positive wasdefined as high risk trisomy report in a case that wassubsequently shown to be without aneuploidy uponinvasive diagnostic confirmation or clinical physicalexamination. Patients without confirmatory diagnosticresults were excluded from calculation of test parameters (sensitivity, specificity, positive perspective value(PPV), and negative perspective value (NPV)).

## Results

### NIPS failure rate

Between 11 October 2016 and 26 June 2019, a total of 32,394 maternal blood samples were obtained for NIPS screening at Findgene from the Changzhi Maternal and Child Health Care Hospital in Shanxi, China. Out of these samples, 33 cases yielded no NIPS results including 24 cases of haemolysis and 9 cases of cancellation. The rate of NIPS failure was 0.1% (33/32394).

### Demographic characteristics of pregnant women undergoing non-invasive prenatal screening (NIPS) for aneuploidies

The demographic characteristics of the remaining 32,361 samples are shown in Table 1. Data from a total of 32,361 cases were included in this study, which mainly consisted of women from Shanxi province, China. The mean maternal age was 31 years, with 22.8% maternal age ≥ 35 years upon delivery. In this cohort, 31,486 cases were single pregnant, 875 cases were twins and 549 cases were *in-vitro* fertilization (IVF). The gestational age (GA) ranges from 11 to 30 weeks, with a mean GA of 18 weeks.

**Table 1:** Demographic characteristics of pregnant women undergoing NIPS for aneuploidies between 11 October 2016 and 26 June 2019.

Maternal age	
Mean age (year)	31
Advanced maternal age > = 35	7,316 (22.8%)
Pregnancies with the age < 35	25,045 (77.2%)
18–20 year	89
20–24 year	4,012
25–29 year	12,260
30–34 year	8,684
35–40 (year)	6,272
>40 (year)	1,044
In Vitro Fertilization sample	549
Twin samples	875
Single pregnant sample	31,486
Gestational age at blood sampling	
Median (week)	18.5
Range (week)	11-30
11 to 15 weeks (n, %)	1,060 (3.3%)
16 to 20 weeks (n, %)	25,651 (79.2%)
20 to 25 weeks (n, %)	4,819 (15%)
26-30 weeks (n, %)	831 (2.5%)

### Efficiency of NIPS for T21/T18/T13

Among the 32,361 confirmed cases who obtained NIPS results, 164 were positive and 32,197cases were negative (Table 1). Pregnancies with the NIPS positive results were recommended for confirmatory invasive prenatal diagnosis using chromosomal microarray analysis. After informed consent, only 114 cases agreed to go through the confirmatory invasive prenatal diagnosis while, 50 cases declined for further diagnosis. Table 2 shows the prenatal diagnosis results. The NIPS sensitive, specificity, positive predictive values and negative predictive values were 100%, 99.91%, 74.78%, 100% respectively.

**Table 2:** Efficiency of NIPS for T21/T18/T13 TP = true positive, FP = false positive, NIPS = noninvasive prenatal screening, PPV = positive predictive value, NPV= Negative predictive value.

Trisomies	TP	FP	FN	Sensitivity	Specificity	PPV	NPV
T21	65	17	0	100%	99.95%	79.27%	100%
T18	17	8	0	100%	99.98%	68%	100%
T13	4	4	0	100%	99.99%	50%	100%
**Total**	**86**	**29**	**0**	**100%**	**99.91%**	**74.78%**	**100%**

### Efficiency of NIPS in high-risk pregnancies and low-risk pregnancies

In present study, the women whose age ≥ 35 (high-risk pregnancies) years old were 7,316 cases. Among 7,316 advanced maternal age women, 29 cases were the NIPS positive and 12 cases were NIPS false positive results of T21/T18/T13 (Table 2). Of all 25,045 cases of the women whose age < 35 (low risk pregnancies), there were 56 cases with NIPS positive results and 17 cases with false positive results. False negative cases were not found after at least 10 months of follow-up. There was no significant difference in test performance between the 7,316 highrisk and 25,045 low-risk subjects (sensitivity, 100% vs. 100% (P >0.05); specificity, 99.96% vs. 99.95% (P > 0.05)). These results suggested that the application of NIPS could significantly reduce the cost of invasive prenatal diagnosis, which had a positive yield of only 0.56% (41/7,316) in high-risk group and 0.29% (73/25,045) in low-risk group.

### Z score and gray zone

Out of 29 false positive cases, 20 cases (69%) had Z score with 3 < Z < 4 and 9 cases (31%) had Z score > 4. In contrast, 85 cases got true positive results of NIPT, 80 (95%) cases got Z > 4, and only 5 cases (5%) cases got Z score ranging from 3 to 4. These results indicated that Z score between 3 and 4 represented a gray zone where most false positive cases resided.

### Follow-up investigation of test negative cases

At the time of writing, all pregnant women included in this series had given birth. To date, among 32,197 NIPS negative cases, no abnormalities were reported through our feedback mechanism.

### Characteristics of single-nucleotide polymorphisms (SNPs) in the Shanxi pregnant genome

As compared to 1000 g Eastern Asian population there were significant difference alleles frequencies in 7,285,418 SNPs variants (p<0.05) (Supplementary 1). Out of these SNPs variants, 19, 293 SNPs variants were recorded by clinivar https://www.ncbi.nlm.nih.gov/clinvar/ including 17,167 benign variants, 67 pathogenic variants, 5 affects variants, 82 associated variants, 408 not provided/other variants, 8 protective variants, 85 risk factor variants, 977 uncertain significance variants, 387 conflicting interpretation of pathogenicity, and 107 drug response variants.

## Discussion

In the past few years, NIPS has been widely used as a powerful screening tool to detect the chromosomal aneuploidies such as T21, T18, and T13 [18]. This prospective study was performed to evaluate the efficiency of NIPS in the Changzhi Maternal and Child Health Care Hospital. The results indicated a significant difference in 7,285,418 SNPs variants of Shanxi pregnant women including 19,293 clinvar and 7,266,125 nonclinvar recorded variants. Our data also showed that the sensitivity, specificity, PPV and NPV were 100%, 99.91%, 74.56% and 100% respectively, which were very competitive compared to earlier reports [12] [19] [20]. When compared to studies in high-risk pregnancies, our results were comparable to those of Qi et al. [21] and Hu et al. [22], showing that the efficacy of NIPS screening was consistent from low-risk to high-risk pregnancies.

In parallel, false positives were analyzed in detail. Some possible reasons of false positives in NIPS had been reported in literature, including low Z scores, fetal pathogenic CNVs and placental mosaicism. Bianchi et al. [23] demonstrated that a Z-score between 2.5 and 4 should be considered as a borderline value [23], and false-positives were likely to occur at borderline Z scores [24]. Indeed, this study indicated that 20 NIPS positive cases (80%) with Z score from 3 to 4 were later confirmed to be false positives (Table 3). The algorithm of NIPS is Z test with the standard normal distribution. If the true negative is judged to be negative by Z 3 (99.87%), the probability of false positive would be 0.13%. Theoretically, the probability of false positives of 0.13% can be accepted. We also developed an algorithm to exclude the effect of maternal CNV and refined the Z-score that can determine fetal aneuploidy. However, biological factors such as fetal pathogenic CNV played an important role in causing the false positives [25]. This study indicated that 2 cases of false positives were related to fetal pathogenic CNV, including a case T13 for 1450KB amplification at 13q12.12 and a case T21 for amplification at 21B, 21I points. Fetal mosaicism had also been reported to cause false results [26]. Moreover, our data also showed that 2 cases of false positive were fetal mosaicism by invasive diagnostic confirmation. These factors must therefore be taken into account when interpreting NIPS results, and post-test genetic counseling should be provided to pregnant women following recommendations, such as those of the National Society of Genetic Counselors' statement.

**Table 3:** The characteristics of Z score in the false and truepositive cases T13 = trisomy 13, T18 = trisomy 18, T21 = trisomy 21.

	Z score	T21	T18	T13	Total
False<br>positive<br>(n=29)	3<Z<4	14	3/7	3/4	20/29 (69%)
	Z>4	4	4/7	1/4	9/29 (31%)
True<br>positive<br>(n=85)	3<Z<4	4	1	0	5/85 (5%)
	Z>4	60	16	4	80/85 (95%)

In present study, the percentage of positive NIPS was 0.51%, which was lower than those reported in other studies, ranging from 1.6 to 3.2% [22] [27]. This was because this study included a large number of low-risk pregnancies (25,046 cases (77.3%)) for NIPS with a mean maternal age of 31 years. Out of the 7,316 advanced maternally aged women, there were only 29 patients (0.39%) showing positive results; Among 25,046 low-risk cases (<35 years), there were 55 cases (0.22%) showing positive results. Although NIPS was often recommended for maternal ages above 35, we demonstrated its efficacy in lowrisk pregnancies. It could significantly reduce the cost of invasive prenatal diagnosis, many of which would be unnecessary. In fact, in this study, application of NIPS significantly reduced the rate of invasive prenatal diagnosis to 0.56% (41/7316) in high-risk group and 0.29% (73/25046) in low-risk group (Table 4). Surprisingly, this study found 44/64 cases (69%) of Down syndrome coming from the low-risk pregnancies (Table 4), this highlighted the value of NIPS in this often-overlooked subgroup.

**Table 4:** Performance of non-invasive prenatal screening (NIPS) in high-risk pregnancies and low-risk pregnancies TP = true positive, FP = false positive, NIPS = noninvasive prenatal screening, T13 = trisomy 13, T18 = trisomy 18, T21 = trisomy 21.

Category	Verified	TP	FP	Sensitivity	Specificity
T13 (<35yrs)	3	2	1	100%	99.99%
T18 (<35yrs)	13	10	3	100%	99.99%
T21 (<35yrs)	56	44	13	100%	99.96%
T13 (>=35yrs)	5	2	3	100%	99.99%
T18 (>=35yrs)	11	7	4	100%	99.99%
T21 (>=35yrs)	25	21	5	100%	99.98%
Low-risk<br>pregnancies<br>(<35yrs)	73	56	17	100%	99.95%
High-risk<br>pregnancies<br>(>=35yrs)	41	29	12	100%	99.96%

## Conclusion

In conclusion, this study showed that NIPS was an effective method for prenatal detection of aneuploidies. It showed comparable results when applied to both high-risk and low-risk pregnancies, and was able to provide valuable information for more cost-effective utilization of invasive diagnostic methods. Although our study provides an authentication of NIPS as effective method to detect the prenatal detection of aneuploidies on the basis of being highly sensitivity and specificity with lower false-positive rates, but still there are some limitations to NIPS and our study. Although NIPS has high sensitivity and specificity, the overall specificity and sensitivity is not uniform for all chromosomes because of the variation in GC content of sequences. Another limitation is that selected patients for NIPS may not reflect a general obstetrical population. Also, our study didn't include the abnormalities determined using ultrasound in comparison to NIPS. Further studies are needed to overcome these issues to narrower the gap between diagnosis and treatment.

## Dodatak

### Data Availability

The data used to support the findings of this study are available from the corresponding author upon request.

### Funding Statement

This research received no external funding.

### Acknowledgment

The authors thank all collaborating medical centers, patients and their families.

### Conflict of interest statement

All the authors declare that they have no conflict of interest in this work.
